# The Effects of Hotel Employees’ Attitude Toward the Use of AI on Customer Orientation: The Role of Usage Attitudes and Proactive Personality

**DOI:** 10.3390/bs15020127

**Published:** 2025-01-24

**Authors:** Peng Wang, Yong Hou

**Affiliations:** School of Economics and Management, Weifang University of Science and Technology, Weifang 262700, China; wangpeng@wfust.edu.cn

**Keywords:** technology acceptance model, perceived usefulness, perceived ease of use, customer orientation, proactive personality

## Abstract

Along with the development and application of artificial intelligence technology, intelligent services are also emerging in the travel industry. Especially in the tourism and hotel industry, many organizations have started to introduce AI to assist their employees. The purpose of this study is to explore the effects of employees’ perceived usefulness and perceived ease of use of AI on customer orientation, and further analyze the mediating role of attitudes toward use and the moderating role of a proactive personality. A questionnaire was administered to hotel employees in Liaoning Province, China, and hypothesis testing was conducted using SPSS 24.0 and AMOS 22.0. It was found that the perceived usefulness and perceived ease of use significantly and positively influenced usage attitudes and customer orientation. The usage attitudes mediated between perceived usefulness/perceived ease of use and customer orientation. Proactive personality moderated the effects of perceived usefulness and perceived ease of use on usage attitudes. This study not only theoretically enriches the research related to technology acceptance modeling, but also practically provides suggestions for hotel managers to manage their employees after the introduction of AI.

## 1. Introduction

Along with the development and application of AI technology, human–robot interaction has become a new working mode (J.-M. Li et al., 2023a, 2024). In the tourism industry, AI is beginning to be used in hotels. Wirtz et al. (2018) define AI as “AI that are systematic, autonomous, and flexible, and at the same time capable of communicating and interacting with businesses and customers and accomplishing certain service tasks”. China’s hotel industry has been growing in its awareness of robotic services, with hotel groups such as Huazhu, Jinjiang, and Yado, as well as brands such as InterContinental, Westin, Marriott, and Wanda, beginning to use AI to provide services. However, some hotels that have purchased delivery AI have deliberately ignored the use of robotic delivery functions because they do not want to break their existing work routines (S. S. Kim et al., 2021). Eventually, the AI were reduced to a single device for promotional parades in hotel lobbies (J.-M. Li et al., 2025; Zhang et al., 2023). Therefore, how to improve the customer orientation of employees under the working mode of human–robot interaction has become an important issue in the field of hotel management.

After reviewing the literature on the factors affecting the customer orientation of hotel employees, we found that leadership style, organizational climate, and relationship with colleagues affect the customer orientation of hotel employees (Al-Ababneh, 2016). Although the above studies have enriched the literature and research on customer orientation to some extent, they have neglected the impact of the introduction of AI on employee customer orientation. In this context, research focusing on the impact of AI on customers should be accompanied by a greater emphasis on understanding the effects of introducing AI on human employees. It is crucial to explore how the integration of AI may influence the roles, responsibilities, and job satisfaction of human staff members within the hotel industry. Hotel employees’ daily work will be supported by technology, and hotels need staff who are capable of effectively utilizing the latest technology to serve customers and meet their expectations (J. J. Li et al., 2019). Beldona et al. (2018) pointed out that in most cases, technology can improve the guest experience by enhancing the efficiency of hotel service delivery. However, the introduction of new technologies often requires employees to invest time in learning and gradually adapting. Parasuraman and Colby (2015) found that, from the perspective of hotel employees, the use of technology-related services within the hotel was perceived as beyond the scope of their duties. In other words, employees perceive the use of technology as additional tasks, leading to some negative emotions.

Therefore, even if hotel managers intend to use AI to provide customers with higher-quality, more efficient, and innovative services, they cannot ignore the negative impact that the use of AI may have on employees. Managers need to have a thorough understanding of employees’ reactions to the use of AI, ensuring that employees maintain a relatively relaxed attitude when using this new technology. Indeed, the customer orientation in a hotel may be influenced by employees’ attitudes toward the use of technology. Therefore, the attitude of hotel employees toward the use of AI is crucial in discussing whether the use of AI can provide high-quality service.

To elucidate the impact of individuals’ acceptance of technology, Davis (1989) proposed the Technology Acceptance Model (TAM), which has been widely applied in various contexts for related research. The Technology Acceptance Model is a model proposed by Davis using the Theory of Reasoned Action to study users’ acceptance of information systems. He initially proposed the Technology Acceptance Model with the primary goal of providing an explanatory framework for the determining factors behind the widespread acceptance of computers. The Technology Acceptance Model proposes two main determining factors: first, perceived usefulness, which reflects the extent to which an individual believes that using a particular system will enhance their job performance. Second, perceived ease of use reflects the extent to which an individual believes that using a particular system is free from effort. The TAM (Technology Acceptance Model) assumes that individuals’ attitudes and intentions to use a system are based on their perceptions of the usefulness or ease of use of a particular technology (Venkatesh & Davis, 2000). Therefore, this article utilizes the TAM (Technology Acceptance Model) to examine the impact of employees’ perceived usefulness and perceived ease of use of AI on their attitudes and intentions to use.

This study has the following contributions. In theory, first, it validates the TAM. Second, this study introduces the personality trait variable of a proactive personality, and explores its moderating role between perceived usefulness, perceived ease of use, and attitude toward usage. Third, this study expands the literature on robot usage in the hotel industry. In practice, first, organizations should take measures to improve hotel employees’ perceptions of the usefulness and ease of use of AI, thereby enhancing employee customer orientation. Second, organizations should take measures to enhance employees’ proactive personality. Third, organizations should take measures to enhance hotel employees’ attitudes toward the use of AI.

The remainder of this paper is organized as follows: Section 2 outlines the theoretical foundation and formulates the hypotheses, drawing on the Technology Acceptance Model and relevant literature. Section 3 describes the methodology, including data collection, measurement tools, and analytical methods. Section 4 presents the results, highlighting hypothesis testing and statistical analysis. Section 5 discusses the findings in the context of theoretical contributions and practical implications. Finally, Section 6 concludes the study by summarizing key insights, identifying limitations, and suggesting directions for future research.

## 2. Theoretical Foundation and Hypothesis Formulation

### 2.1. Theoretical Foundation

The TAM, first proposed by Davis in 1986, is an improvement upon the Theory of Reasoned Action (TRA) (Marangunić & Granić, 2015). It introduces two concepts as determinants of the intention to use and attitude toward the use of information technology. The first concept is perceived usefulness (PU), defined as the degree to which a user believes that using a particular technology or system will enhance his or her job performance. The second concept is perceived ease of use (PEOU), defined as the degree to which a user expects that using a particular technology or system will be free of effort (Davis, 1989). Attitude toward use refers to the user’s favorability or unfavorability toward the use of a particular technology or system. The intention to use is the direct determinant of a user’s decision to use a technology or system, serving as a necessary prerequisite for actual behavior (Baskaran et al., 2020).

### 2.2. Perceived Usefulness and Ease of Use in Relation to Customer Orientation

Perceived usefulness and perceived ease of use, as two crucial factors in the technology acceptance model, are commonly employed to explain user attitudes and behaviors toward the acceptance of new technologies. They have a positive impact on the customer orientation of hotel employees (Marangunić & Granić, 2015).

First, perceived usefulness refers to the subjective assessment by users of whether the use of technology results in practical value and benefits. For the use of AI, if hotel employees perceive that the AI can provide assistance, alleviate workload, and enhance work efficiency, among other practical values in their work. Therefore, they may be more willing to accept and use this technology (Choi et al., 2020; J.-M. Li et al., 2024). For instance, AI can handle guest reception at hotel front desks, provide information assistance, deliver meals, and thereby alleviate the workload of staff, enhance service efficiency, and offer an improved service experience (Lukanova & Ilieva, 2019). If employees believe that AI can genuinely enhance their work effectiveness, they will be more inclined to use it actively, consequently positively influencing the customer orientation (Fuentes-Moraleda et al., 2020).

Second, perceived ease of use refers to the subjective assessment by users of whether the technological usage process is convenient, simple, and easy to operate (Venkatesh & Davis, 1996; Wu et al., 2023b). If hotel employees perceive using AI as a convenient, simple, and user-friendly experience, they might be more inclined to leverage AI to enhance customer orientation (Chien et al., 2019). For example, if the interface design of a service robot is simple and intuitive, with easy-to-use functionalities, employees can quickly grasp how to use it. Through seamless operations, the robot can assist employees in tasks such as delivering and collecting items or facilitating check-out, requiring minimal training and learning. This can significantly enhance customer orientation in the hotel industry (J.-M. Li et al., 2023a; Seo & Lee, 2021). When employees perceive AI as both useful and easy to use, they are likely to be more willing to actively engage with them. Moreover, during the utilization of AI, employees can fully leverage their functionalities, leading to an enhancement in customer orientation. Employees are more inclined to actively utilize it and fully leverage its functionalities during usage, thereby enhancing customer orientation (J.-M. Li et al., 2024). Therefore, this study proposes the following hypotheses:

**H1:**  *The perception of usefulness positively influences customer orientation.*

**H2:**  *The perception of ease of use positively influences customer orientation.*

### 2.3. The Perception of Usefulness and Ease of Use Is Associated with the Attitude Toward Usage

The perception of usefulness and perceived ease of use may be closely related to employees’ attitudes towards the use of AI. Perceived usefulness refers to users’ cognitive evaluation of technology, indicating their belief that using the technology will bring tangible benefits and improve job performance (Anton et al., 2014). When users perceive that a technology has practical value and benefits for their work or life, they are more likely to hold a positive attitude toward that technology (J.-M. Li et al., 2023a). For example, when studying hotel staff attitudes toward the use of AI, if employees believe that AI can enhance their work efficiency, reduce workload, or provide a better service experience, they would perceive the technology as useful and likely hold a positive attitude toward it (Venkatesh & Davis, 2000).

Second, the perceived ease of use may also influence employees’ attitudes toward the use of AI. Perceived ease of use refers to users’ cognitive evaluation of the process of using technology, encompassing factors such as the learning curve, ease of operation, and the user-friendliness of the interface during use (Wu et al., 2023b). When users perceive that the process of using technology is simple, user-friendly, and does not entail excessive learning or operational costs, they are more likely to hold a positive attitude towards the technology. In the study of hotel employees’ attitudes toward the use of AI, if employees perceive that the interface design of the service robot is intuitively simple, with operations resembling common household appliances, they can quickly grasp its usage without requiring extensive training and learning. In such cases, they are likely to consider the technology as user-friendly and hold a positive attitude toward it.

Therefore, according to the TAM (Technology Acceptance Model), when hotel employees perceive practical benefits and improved work performance value in AI, and find the technological usage process to be straightforward and user-friendly, they are likely to hold a positive attitude toward AI (Marangunić & Granić, 2015; Venkatesh & Davis, 2000). This is because they consider the technology to be useful and easily applicable in their work, leading them to hold a positive attitude towards it (Baskaran et al., 2020). In summary, this study proposes the following hypotheses:

**H3:**  *The perceived usefulness positively influences the attitude toward usage.*

**H4:**  *The perceived ease of use positively influences the attitude toward usage.*

### 2.4. The Attitude Toward Usage and Customer Orientation

The TAM suggests that the behavioral intention of users towards adopting new technology is influenced by their attitude toward its use (Menant et al., 2021). In the hotel industry, employees’ attitudes toward the use of AI may similarly affect the ultimate customer orientation.

First, when employees have a positive attitude toward AI, they are more likely to exhibit positive behaviors in actual operations (Naneva et al., 2020). They may be more inclined to use AI and approach interactions with them with a positive attitude. This positive attitude and behavior often lead employees to pay more attention to customer orientation and exert additional efforts to provide high-quality services (Ivanov et al., 2018a).

At this point, AI may be able to complete tasks more smoothly, provide more efficient and accurate services, thereby positively impacting customer orientation (Ivanov et al., 2018b). Second, when employees have a positive attitude toward the technology they are using, they are more inclined to actively address issues in the technology’s use process, rather than treating problems with indifference or negativity (Said et al., 2023; Wu et al., 2023a). This means they will pay greater attention to addressing the difficulties and challenges in the technology usage process, thereby improving the effectiveness of technology utilization and customer orientation. For example, in the case of hotel employees using AI, if they have a positive attitude toward the AI, they will be more willing to actively address any technical issues that may arise during the use of the AI, ensuring the smooth operation of the AI and thereby enhancing the customer orientation. In summary, we believe that a positive attitude toward technology usage can lead employees to invest more effort and resources, be more willing to cooperate and support technology usage, and actively address issues in the technology usage process (J.-M. Li et al., 2023a; Venkatesh & Davis, 2000). This allows for the better integration of AI into service work, ultimately enhancing customer orientation. We propose the following hypothesis:

**H5:**  *Attitude toward usage positively impacts customer orientation.*

### 2.5. The Mediating Role of Usage Attitude

Based on the above hypothesis, we further believe that usage attitude may mediate the impact of perceived usefulness on customer orientation and the impact of perceived ease of use on customer orientation. Specifically, when employees perceive technology as useful, they are more likely to demonstrate a positive willingness to use it, thereby increasing their involvement and performance in the service process, which, in turn, further affects customer orientation. In this case, willingness to use acts as a mediating variable between perceived usefulness and customer orientation (J.-M. Li et al., 2023a).

When employees perceive technology as easy to use, they are also more likely to demonstrate a positive willingness to use it, thereby further influencing their involvement and performance in the service process, ultimately enhancing customer orientation (Luo et al., 2021). When employees perceive technology as easy to use, they are more willing to utilize it and exhibit a more positive attitude and level of commitment during its usage, thereby positively influencing customer orientation (Belanche et al., 2021). For instance, when hotel employees perceive AI as highly user-friendly, they are likely to be more willing to use this AI and exhibit a more positive attitude during their usage, consequently enhancing customer orientation (J.-M. Li et al., 2023a). Therefore, this study proposes the following hypotheses:

**H6:**  *Attitude toward the use of AI by hotel employees mediates the relationship between perceived usefulness and customer orientation.*

**H7:**  *Attitude toward the use of AI by hotel employees mediates the relationship between perceived ease of use and customer orientation.*

### 2.6. The Moderating Role of Proactive Personality

As a personality trait, proactive personality refers to an individual’s tendency to exhibit spontaneous, proactive, and self-initiated behavior and decision-making (Fuller & Marler, 2009). Employees with high proactive personalities tend to challenge the status quo and actively seek to improve the current situation or create new ones, rather than passively adapting to the current circumstances (McCormick et al., 2019). Previous research has shown that proactive employees take initiative to improve situations rather than accept them, and they attempt to find new technological features, innovative applications, and engage in creative integration of technology and task knowledge (Erdogan & Bauer, 2005).

In the study of hotel employees’ attitudes toward AI, this research introduces a proactive personality as a moderating variable. This study delves into the impact of employees’ perceived usefulness and perceived ease of use on their attitude toward usage, aiming to gain a deeper understanding of the mechanism behind the formation of employees’ attitudes toward AI (J.-M. Li et al., 2023a).

First, a proactive personality may positively enhance the moderating role of perceived usefulness on attitude toward usage. Employees with proactive personalities typically possess greater self-motivation and initiative. They may be more attentive to the potential value and advantages of technology and more willing to proactively explore new technologies (Major et al., 2006). Therefore, when these employees perceive the usefulness of AI, they may be more likely to develop a positive attitude toward usage, exhibiting higher willingness to use and proactive behavior (McCormick et al., 2019). They may be more inclined to proactively explore and utilize AI, thereby enhancing the positive impact of perceived usefulness on attitude towards usage.

Second, a proactive personality may positively enhance the moderating role of perceived ease of use on attitude towards usage. Employees with proactive personalities typically possess higher self-efficacy and self-drive, and they may be more willing to confront technological challenges and overcome difficulties in technology usage (Crant & Bateman, 2000). When these employees perceive the ease of use of AI, they may be more likely to develop a positive attitude toward usage, exhibiting higher willingness to use and proactive behavior. They may be more inclined to proactively try and master the ways of using AI, thereby strengthening the positive impact of perceived ease of use on attitude toward usage (T.-Y. Kim et al., 2009). Therefore, this study proposes the following hypotheses:

**H8:**  *Proactive personality moderates the relationship between perceived usefulness and attitude toward usage, with higher levels of a proactive personality among employees being more likely to result in an increased willingness to use perceived usefulness, and vice versa.*

**H9:**  *Proactive personality moderates the relationship between perceived ease of use and attitude toward usage, with higher levels of a proactive personality among employees being more likely to result in an increased willingness to use perceived ease of use, and vice versa.*

**Figure 1:**
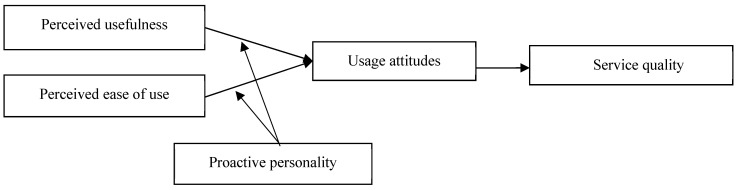
Research model.

In summary, the model diagram for this study is depicted in Figure 1.

## 3. Methods

### 3.1. Procedure

Hotel industry data in Liaoning Province shows that the province received more than 150 million domestic tourists in 2022, with a hotel occupancy rate of more than 60%. In addition, major cities in Liaoning province, such as Shenyang and Dalian, have more than 2000 star-rated hotels to meet diversified market demand. In recent years, the government has been actively promoting the development of the tourism industry by upgrading infrastructure and service levels, which has created a favorable environment for further growth of the hotel industry. In addition, Liaoning Province, an important economic and tourism center in Northeast China, is rich in history, culture and natural resources. According to the data, the hospitality industry in Liaoning Province has continued to grow in recent years, attracting a large number of domestic and international tourists. The reasons for choosing Liaoning Province include its unique geographic location, development potential, and policy support for the hospitality industry, all of which provide rich background and empirical data for the study. Therefore, this paper selected hotels in Liaoning Province, China, as a sample.

To test the hypotheses proposed in this study, data were collected from employees of 15 hotels in Shenyang, Liaoning Province, China, between January and February 2023. To ensure the accuracy of responses, hotels were selected based on whether their employees used AI in their daily work. Hotels that did not use AI to assist employees were excluded from the sample. The survey was distributed with the assistance of middle-level managers from the relevant hotels, who were contacted through guidance from the supervising instructor and other social connections.

To mitigate the potential adverse effects of common method bias on the study results, data were collected at two separate time points. At Time 1 (15 January 2023), the first questionnaire was distributed, primarily measuring perceived usefulness, perceived ease of use, and proactive personality. At Time 2 (15 February 2023), the second questionnaire was distributed, primarily measuring attitude toward usage, customer orientation, and demographic variables. In the first round of data collection, a total of 430 questionnaires were collected. After removing ineligible questionnaires, 401 valid responses were obtained. In the second round of data collection, 389 questionnaires were collected, and after eliminating ineligible ones, 377 valid responses were retained. In the data screening process, invalid samples were excluded based on several criteria: (1) incomplete responses—questionnaires with missing data for key variables, such as usage attitudes or perceived usefulness, were deemed invalid; (2) abnormal response patterns—responses showing a lack of variability (e.g., selecting the same option for all items) or nonsensical answers (e.g., unrealistic age or work experience) were removed; (3) duplicated submissions—identified through IP addresses or timestamps to ensure each respondent contributed only once; (4) mismatch with the target population—respondents who were not hotel employees or whose hotels did not use AI were excluded; and (5) unrealistic completion times—questionnaires completed too quickly to reflect thoughtful responses were eliminated. These criteria ensured the reliability and relevance of the final dataset.

An analysis of the 377 valid responses reveals the following demographic characteristics of the sample. Regarding gender, there were 199 females, constituting 52.79% of the total sample, and 178 males, making up 47.21% of the total sample. In terms of educational attainment, 13 employees had a junior high school education or below, accounting for 3.45% of the sample. Twenty-two employees had a high school education, comprising 5.84% of the sample. Thirty-four employees had completed vocational education, representing 9.02% of the sample. Fifty-eight employees held an associate degree, constituting 15.38% of the sample. Finally, 250 employees had a bachelor’s degree or higher, making up 66.31% of the total sample.

### 3.2. Measurement

To ensure the reliability and validity of the questionnaire, all scales used in this study are well-established and widely used. All scales were rated using a Likert five-point scale, where ‘1’ to ‘5’ indicated ‘strongly disagree’ to ‘strongly agree’. Since the data were collected in China, the questionnaire was distributed in Chinese as the primary language. A rigorous “translate-back-translate” process was conducted by all the authors in designing the questionnaire to ensure that the meaning of the Chinese questionnaire was consistent with the original English scale. The original English questionnaire was first translated into Chinese by a bilingual translator who was fluent in both English and Chinese. This translator was familiar with the subject matter and the context of the research. Once the Chinese version was finalized, a second bilingual translator, who was not involved in the initial translation, translated the Chinese version back into English. This step aimed to ensure that the meaning of the items in the Chinese questionnaire matched the original English version. After the back-translation, the research team compared the back-translated English version with the original English questionnaire to identify any discrepancies or differences in meaning. Any inconsistencies or ambiguities in the translation were discussed and resolved through consensus among the authors and translators. Finally, a pre-test of the Chinese version was conducted with a small sample of participants to assess clarity and comprehension. Based on their feedback, further revisions were made to ensure that the Chinese version accurately reflected the original content and was culturally appropriate. Please see Appendix A for the specific questions items of the scale.

#### 3.2.1. Perceived Usefulness

The measurement of this variable utilized a scale developed by Sun et al. (2020), consisting of 6 items such as “Using AI has improved my job performance’ and ‘Overall, I find AI to be useful in my work”. The Cronbach’s α for this scale was 0.82.

#### 3.2.2. Perceived Ease of Use

The measurement of this variable utilized a scale developed by Sun et al. (2020), consisting of 5 items such as “I find it easy to make AI do what I want them to do” and ‘Learning to operate AI is easy for me’. The Cronbach’s α for this scale was 0.88.

#### 3.2.3. Attitude Toward Usage

The measurement of this variable utilized a scale developed by M. Kim and Qu (2014), consisting of 4 items such as “I enjoy using hotel AI” and “I intend to continue using AI”. The Cronbach’s α for this scale was 0.92.

#### 3.2.4. Proactive Personality

The measurement of this variable utilized a scale developed by Ling et al. (2016), consisting of 6 items such as “To improve performance, I often perform tasks beyond my job scope” and “If I see something I don’t like, I will overcome it”. The Cronbach’s α for this scale was 0.84.

#### 3.2.5. Customer Orientation

The measurement of this variable utilized a scale developed by Ling et al. (2016), consisting of 2 items such as “I am very enthusiastic in assisting customers” and “I always communicate with customers proactively and warmly”. The Cronbach’s α for this scale was 0.91.

#### 3.2.6. Control Variables

This study also selected gender, age, education level, and years of work experience as the control variables. For gender and education level, gender was coded as 0 for females and 1 for males, while education level was coded as 1 for junior high school or below, 2 for high school, 3 for vocational education, 4 for associate degree, and 5 for bachelor’s degree or higher. The age and years of work experience are continuous variables, reflected as open-ended questions in the questionnaire, where hotel employees directly provided their age and years of work experience.

## 4. Result

### 4.1. Common Method Bias

This study conducted a test for common method bias using the Harman’s single-factor test. Using SPSS 24.0 software, all variables were simultaneously subjected to factor analysis. The results revealed that the variance explained by the first common factor was 24.21%, which did not exceed the critical threshold of 40%. This suggests that the issue of common method bias in the data of this study is not severe, allowing for further analysis (Podsakoff et al., 2024).

### 4.2. Confirmatory Factor Analysis

This study conducted confirmatory factor analysis using AMOS 22.0 software, and the results are presented in Table 1. As shown in the table, the five-factor model exhibited the best fit compared to other factor models. Therefore, the data in this study passed the validity test.

### 4.3. Descriptive Statistical Analysis

This study conducted a descriptive statistical analysis using SPSS 24.0, and the results are presented in Table 2. As shown in the table, perceived usefulness and perceived ease of use are significantly positively correlated with attitude towards usage and customer orientation. Attitude toward usage and customer orientation are also significantly positively correlated. These findings provide initial support for the hypotheses in this study.

In addition, the factor loadings for each question item and the average variance extracted for each variable are reported in Table 3. As can be seen from Table 3, the factor loadings for each question item and the average variance extracted for each variable reached the criterion values.

### 4.4. Hypothesis Testing

This study conducted hypothesis testing using SPSS 24.0, and the results are presented in Table 4. As shown in Table 4, perceived usefulness (B = 0.30, SE = 0.06, *p* < 0.001) and perceived ease of use (B = 0.27, SE = 0.05, *p* < 0.001) significantly positively impact customer orientation, confirming H1 and H2. Perceived usefulness (B = 0.39, SE = 0.07, *p* < 0.001) and perceived ease of use (B = 0.30, SE = 0.05, *p* < 0.001) significantly positively impact attitude toward usage, confirming H3 and H4. Attitude toward usage (B = 0.12, SE = 0.04, *p* < 0.01) significantly influences customer orientation, confirming H5.

This study employs the three-step method proposed by Baron and Kenny (1986) to test the mediating role of “usage attitude” between perceived usefulness/perceived ease of use and customer orientation. The first step examines the direct effect of the independent variable (perceived usefulness/perceived ease of use) on the dependent variable (customer orientation). The second step verifies the significant effect of the independent variable on the mediator (usage attitude). The third step includes both the independent and mediating variables in the model to test the mediator’s effect on the dependent variable while observing whether the direct effect of the independent variable weakens. The results indicate that “usage attitude” serves as a partial mediator for both perceived usefulness and perceived ease of use, supporting the hypotheses and validating the applicability of the Technology Acceptance Model. Therefore, H6 and H7 are confirmed. We further tested the mediating role of usage attitude using Bootstrap method (Preacher & Hayes, 2004). The results showed that the 95% confidence interval for the mediating role of usage attitude between perceived usefulness and customer orientation was [0.11, 0.45]. The 95% confidence interval for the mediating role of usage attitude between perceived ease of use and customer orientation was [0.08, 0.39]. Since the confidence interval does not include 0, H6 and H7 were again validated.

The mediating role of usage attitude highlights its importance as a mechanism through which perceived usefulness and perceived ease of use enhance customer orientation. While the direct effects remain significant, suggesting partial mediation, this aligns with prior research on the Technology Acceptance Model (e.g., Venkatesh & Davis, 2000), which emphasizes the interplay between direct and indirect pathways. The bootstrapping results further substantiate this by providing confidence intervals that exclude zero, ensuring robust evidence of mediation.

**Figure 2:**
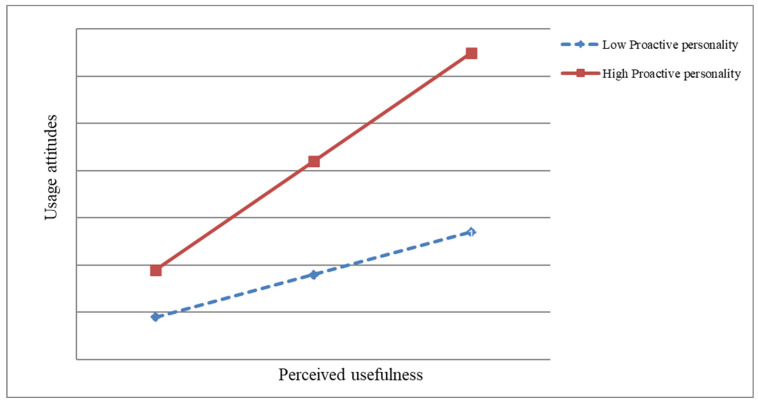
Proactive personality moderation between perceived usefulness and usage attitude.

Subsequently, the test for the moderating role of proactive personality was conducted, and the results are presented in Table 5. As shown in Table 5, when controlling for covariates and including the independent variables, the moderating variable, and their interaction terms simultaneously in the model, the interaction terms significantly affect attitude toward usage. Therefore, proactive personality moderates the effect of perceived usefulness on attitude toward usage, confirming H8. Subsequently, this study plotted the moderating role of proactive personality between perceived usefulness and attitude toward usage, as shown in Figure 2. Based on Figure 2, which posits that “proactive personality moderates the relationship between perceived usefulness and attitude towards usage”. Proactive personality acts as a moderating variable, with higher levels of proactive personality leading to a stronger positive relationship between perceived usefulness and attitude toward usage. Specifically, employees with a high proactive personality are more likely to show an increased willingness to use something perceived as useful, whereas employees with a low proactive personality exhibit a weaker relationship between perceived usefulness and usage attitude. This suggests that proactive individuals are more responsive to the perceived benefits of a tool, enhancing their attitude toward its use.

**Figure 3:**
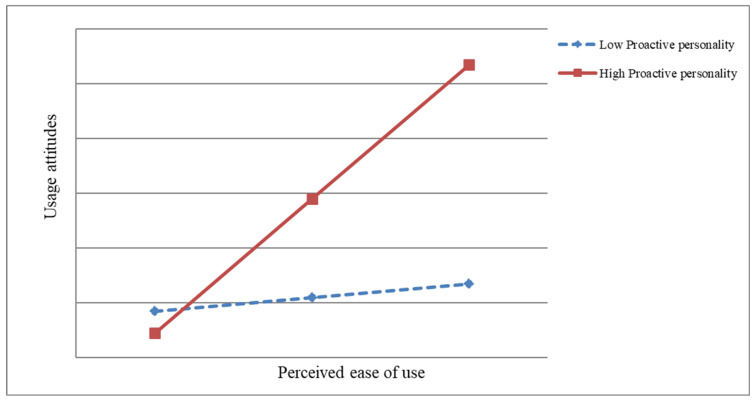
Proactive personality moderation between perceived ease of use and usage attitude.

As shown in Table 5, when controlling for covariates and including the independent variables, the moderating variable, and their interaction terms simultaneously in the model, the interaction terms significantly affect attitude toward usage. Therefore, proactive personality moderates the effect of perceived ease of use on attitude toward usage, confirming H9. Subsequently, this study plotted the moderating role of proactive personality between perceived ease of use and attitude toward usage, as shown in Figure 3. Based on Figure 3, which suggests that “proactive personality moderates the relationship between perceived ease of use and attitude towards usage”, the moderating effect diagram illustrates how proactive personality influences this relationship. Employees with a high proactive personality show a stronger positive relationship between perceived ease of use and attitude toward usage, meaning they are more likely to develop a positive attitude as the technology becomes easier to use. On the other hand, employees with a low proactive personality exhibit a weaker relationship, showing less change in attitude despite perceived ease of use. Thus, proactive individuals are more responsive to ease of use when forming attitudes toward adopting new technology.

## 5. Discussion

This study verified the impact of perceived usefulness and perceived ease of use on customer orientation of hotel employees. Past research has focused on exploring the impact of perceived usefulness and perceived ease of use in the consumer domain (Holden & Karsh, 2010; King & He, 2006). The present study builds on this foundation and introduces it to the hospitality domain. In addition, this study validates the moderating role of proactive personality. Past research has focused on exploring the role of proactive personality in traditional work situations. The present study further validates and extends the research on proactive personality by introducing it into a human–computer collaborative work situation (Crant et al., 2016; J.-M. Li et al., 2023a).

### 5.1. Theoretical Contributions

This study holds theoretical significance in several aspects. First, it validates the Technology Acceptance Model (TAM). Since its inception, TAM has been widely validated in various fields (Holden & Karsh, 2010; King & He, 2006). However, the current use of the Technology Acceptance Model is predominantly concentrated in the field of marketing, such as in the context of live streaming on the internet. Compared to the marketing field, the use of this model in the organizational context has been relatively limited (Legris et al., 2003). This study introduces the Technology Acceptance Model into the hotel industry, exploring the impact of robot usage on employee customer orientation. In doing so, it contributes to the validation and expansion of the Technology Acceptance Model within the context of the hospitality industry.

Second, this study introduces the personality trait variable of proactive personality and explores its moderating role between perceived usefulness, perceived ease of use, and attitude toward usage. The results indicate that when hotel employees possess a strong proactive personality, the impact of perceived usefulness and perceived ease of use on attitude toward usage becomes stronger. Past research has primarily explored the role of proactive personality in traditional organizational management contexts (Crant et al., 2016; J.-M. Li et al., 2023a; Wu et al., 2024). However, with the rapid advancement of artificial intelligence, the question of whether employees with proactive personality traits can adapt well to intelligent devices has become an important research issue (J.-M. Li et al., 2023b).This study builds upon previous research by exploring the moderating role of proactive personality. This finding, to a certain extent, extends the relevant research on proactive personality and holds value for future investigations into the impact of personality traits on the customer orientation of hotel employees.

Third, this study expands the literature on robot usage in the hotel industry. With the rapid advancement of technology, organizations have increasingly introduced intelligent machines to assist employees in their work. For instance, many hotels have already begun to introduce AI to assist employees in performing some basic tasks (J.-M. Li et al., 2024; J.-M. Li et al., 2023a; Lukanova & Ilieva, 2019). Therefore, research on human–machine collaboration in organizational contexts has garnered significant attention from scholars. Currently, research in this field primarily focuses on the impact of the use of artificial intelligence and algorithms on employees and organizations (J.-M. Li et al., 2023a; Lukanova & Ilieva, 2019). This study, taking the perspective of AI and selecting the hotel industry as its research subject, to some extent enriches the relevant literature on the use of AI in the hospitality industry.

### 5.2. Practical Implications

This study has practical implications in the following areas. Organizations should tailor their strategies to different hotel categories to maximize the benefits of AI. For luxury hotels, AI can be leveraged to enhance personalized guest experiences by facilitating bespoke services such as tailored recommendations, seamless concierge support, and real-time response to high-end client needs. This can create a sophisticated and technologically advanced environment that aligns with guest expectations for premium services. For budget hotels, AI can be deployed to streamline operational efficiency and reduce costs while maintaining consistent customer orientation. Applications such as automated check-ins, room allocation, and virtual customer support can help these hotels cater to cost-conscious travelers efficiently and reliably. This ensures that guests receive prompt and standardized service without compromising affordability. By addressing the unique requirements of luxury and budget hotels, AI adoption strategies can be made more effective, ultimately improving customer orientation and customer satisfaction across different market segments.

Second, organizations should take measures to enhance employees’ proactive personality. For example, they should conduct relevant training: training on a proactive personality can be conducted for hotel employees, teaching them how to proactively identify opportunities for improvement in the hotel, how to confidently express their opinions and ideas, and how to take action based on the hotel’s operational situation. They should establish incentive mechanisms: a system of incentives can be established to motivate hotel employees to identify improvement opportunities, take action, and proactively solve problems in a timely manner, such as offering rewards or compensation. They should increase communication opportunities: regularly hold work meetings to provide hotel employees with opportunities to communicate with managers or other hotel executives, allowing them to showcase their ideas and promptly offer suggestions for improvement, thereby enhancing their proactivity. They should organize team activities: hotels can organize team-building activities for employees, such as participating in sports competitions or attending lectures. These activities can strengthen team cohesion, stimulate proactivity, and cultivate teamwork spirit among employees.

Third, organizations should take measures to enhance hotel employees’ attitudes toward the use of AI. For example, they should establish a unified training system: hotels should establish a unified training system to ensure that every employee can master the use of AI and improve their skills. They should organize activities: regular employee activities, such as robot technology training and seminars, should be organized to keep employees informed about the latest robot technologies and future developments, fostering a positive attitude toward robot use among employees. They should provide rewards: for hotel employees who use AI, additional incentives and rewards should be provided to motivate employees’ attitudes toward robot use. They should provide technical support: to maximize the effectiveness of AI, hotels should provide technical support to help employees address any issues they encounter while using AI, thereby improving their technical skills and knowledge.

### 5.3. Research Limitations and Future Research Directions

This study has several limitations that should be considered. First, the cultural and geographic specificity of the data warrants attention. The research was conducted in Liaoning Province, China, a region with unique economic, cultural, and policy characteristics. These factors may influence hotel employees’ attitudes and behaviors toward AI in ways that differ from other regions or countries. For instance, the rapid adoption of AI in China’s hospitality industry might reflect local technological policies and cultural receptiveness to innovation, which may not generalize to regions with slower technological adoption or differing employee expectations. To address this limitation, future studies should expand the geographic scope to include multiple provinces in China and international contexts. Comparative research in culturally distinct regions, such as Western or other Asian countries, could help evaluate the applicability of these findings in diverse hospitality markets.

Second, while this study focuses on the Technology Acceptance Model, alternative theoretical frameworks such as Resource Conservation Theory or Self-Determination Theory could provide additional insights into the dynamics of AI adoption. Exploring these perspectives may reveal novel moderating variables or mechanisms influencing customer orientation.

Third, the moderating role of proactive personality was examined, but other personality traits, such as openness to experience or cultural dimensions (e.g., power distance), may also influence the relationship between AI acceptance and customer orientation. Additionally, organizational factors like workplace culture or leadership style could be explored as moderators in future research. By addressing these limitations, future research can better understand the broader implications of AI adoption in hospitality and provide more generalizable insights.

## 6. Conclusions

This study is based on the Technology Acceptance Model and investigates the use of AI by employees in the hotel industry. The research findings indicate that perceived usefulness and perceived ease of use significantly and positively affect attitude and customer orientation. Attitude serves as a mediator between perceived usefulness and customer orientation, as well as between perceived ease of use and customer orientation. Proactive personality moderates the impact of perceived usefulness and perceived ease of use on attitude, meaning that employees with a stronger proactive personality are more influenced by perceived usefulness and perceived ease of use in shaping their attitude.

## Figures and Tables

**Table 1:** Confirmatory factor analysis results.

Model	χ^2^/df	RMSEA	CFI	GFI	NFI	IFI	TLI	RFI
Single-factor model (1+2+3+4+5)	6.980	0.116	0.559	0.516	0.523	0.561	0.533	0.494
Two-factor model (1+2+3+4,5)	5.297	0.098	0.684	0.602	0.639	0.685	0.664	0.616
Three-factor model (1+2+3,4,5)	3.865	0.080	0.790	0.683	0.737	0.791	0.776	0.720
Four-factor model (1+2,3,4,5)	2.416	0.056	0.897	0.801	0.837	0.897	0.889	0.825
Five-factor model (1,2,3,4,5)	1.133	0.017	0.990	0.928	0.924	0.990	0.990	0.918

Note: 1 represents perceived usefulness, 2 represents perceived ease of use, 3 represents usage attitude, 4 represents customer orientation, and 5 represents proactive personality.

**Table 2:** Correlation coefficient table.

Variable	Mean	SD	1	2	3	4	5	6	7	8	9
1. Gender	1.47	0.50	1								
2. Age	29.57	5.59	0.06	1							
3. Education Level	4.35	1.08	0.05	0.01	1						
4. Years of Work Experience	6.36	4.90	0.06	0.94 ***	−0.02	1					
5. Proactive personality	3.76	0.84	0.08	0.05	0.01	−0.01	1				
6. Perceived Ease of Use	2.18	0.90	−0.03	0.02	−0.05	0.03	0.29 ***	1			
7. Perceived Usefulness	3.65	0.67	0.03	0.07	−0.03	0.06	0.31 ***	0.17 ***	1		
8. Usage Attitude	3.63	1.02	−0.03	0.07	0.01	0.05	0.29 ***	0.31 ***	0.30 ***	1	
9. Customer orientation	3.93	0.87	0.05	0.04	0.02	0.03	0.32 ***	0.32 ***	0.28 ***	0.28 ***	1

*** *p* < 0.001.

**Table 3:** The results of factor loadings and average variance extracted.

Variable	Item	Factor Loadings	Average Variance Extracted	Composite Reliability
Proactive personality	A1	0.83	0.71	0.94
	A2	0.82		
	A3	0.89		
	A4	0.81		
	A5	0.82		
	A6	0.88		
Perceived ease of use	B1	0.84	0.79	0.95
	B2	0.87		
	B3	0.91		
	B4	0.89		
	B5	0.93		
Perceived usefulness	C1	0.82	0.69	0.93
	C2	0.86		
	C3	0.83		
	C4	0.91		
	C5	0.79		
	C6	0.76		
Usage attitude	D1	0.83	0.71	0.91
	D2	0.81		
	D3	0.90		
	D4	0.82		
Customer orientation	E1	0.89	0.77	0.87
	E2	0.86		

**Table 4:** Mediation analysis results.

Model	Customer Orientation		Usage Attitude		Customer Orientation	
	B	SE	B	SE	B	SE
Education Level	0.01	0.04	0.00	0.04	0.01	0.04
Gender	0.05	0.08	−0.12	0.10	0.07	0.08
Age	0.01	0.02	0.03	0.03	0.01	0.02
Years of Work Experience	−0.01	0.02	−0.02	0.03	0.01	0.02
Perceived Ease of Use	0.27 ***	0.05	0.30 ***	0.05	0.23 ***	0.05
Perceived Usefulness	0.30 ***	0.06	0.39 ***	0.07	0.25 ***	0.06
Usage Attitude					0.12 **	0.04
*R* ^2^	0.15		0.17		0.17	
Δ*R*^2^	0.14		0.16		0.16	

** *p* < 0.01 *** *p* < 0.001.

**Table 5:** Moderation effect of proactive personality.

Model	M1		M2		M3	
	B	SE	B	SE	B	SE
Education Level	0.02	0.05	0.02	0.05	0.02	0.05
Gender	−0.11	0.10	−0.13	0.10	−0.13	0.10
Age	0.03	0.03	0.01	0.03	0.01	0.03
Years of Work Experience	−0.02	0.03	−0.01	0.03	−0.01	0.03
Perceived Usefulness			0.37 ***	0.08	0.32 ***	0.08
Perceived Ease of Use			0.35 ***	0.06	0.27 ***	0.06
Proactive Personality			0.26 ***	0.06	0.24 ***	0.06
Int1					0.14 *	0.07
Int2					0.22 ***	0.06
*R* ^2^	0.12		0.16		0.18	
Δ*R*^2^	0.09		0.14		0.14	

* *p* < 0.05 *** *p* < 0.001. Dependent variable: Usage attitudes Int1: Perceived Usefulness × Proactive Personality; Int2: Perceived Ease of Use × Proactive Personality.

## Data Availability

The data that support the findings of this study are available from the first author upon reasonable request. We were unable to share the data due to the participant’s request.

## Appendix A

***Items measuring proactive personality***

1. In order to improve my performance, I often perform tasks that are outside the scope of my job.
2. If I see something I don’t like, I get over it.
3. I feel very happy when the ideas I come up with become reality.
4. I am always looking for new ways to improve my life.
5. I am always looking for better ways to do things.
6. I am willing to believe in my ideas, even against the opposition of others.

***Items measuring perceived ease of use***

1. I find it easy to get the hotel technology to do what I want it to do.
2. Learning to operate the hotel technology is easy for me.
3. Overall, I find the hotel technology easy to use.
4. It is easy for me to remember how to perform tasks using hotel technology.
5. Usage of the hotel technology is understandable.

***Items measuring perceived usefulness***

1. Using hotel technology increases my productivity.
2. Using hotel technology enhances my effectiveness on the job.
3. Overall, I find hotel technology useful in my job.
4. Using hotel technology makes it easier to do my job.
5. Hotel technology enables me to accomplish tasks more quickly.
6. Using hotel technology improves my job performance.

***Items measuring usage attitude***

1. All things considered, using AI is a good idea
2. All things considered, using AI is advisable
3. All things considered, using AI is pleasant
4. I enjoy using AI

***Items measuring customer orientation***

1. I am very passionate about helping customers.
2. I am always proactive and enthusiastic with my customers.
